# *Echinococcus multilocularis* Detection in Live Eurasian Beavers (*Castor fiber*) Using a Combination of Laparoscopy and Abdominal Ultrasound under Field Conditions

**DOI:** 10.1371/journal.pone.0130842

**Published:** 2015-07-13

**Authors:** Róisín Campbell-Palmer, Jorge Del Pozo, Bruno Gottstein, Simon Girling, John Cracknell, Gerhard Schwab, Frank Rosell, Romain Pizzi

**Affiliations:** 1 Veterinary Department and Conservation Programmes, Royal Zoological Society of Scotland, Edinburgh, United Kingdom; 2 Department of Veterinary Pathology, Royal (Dick) School of Veterinary Studies, University of Edinburgh, Edinburgh, United Kingdom; 3 Institute of Parasitology, Vetsuisse Faculty, University of Bern, Bern, Switzerland; 4 Longleat Safari and Adventure Park, Warminster, United Kingdom; 5 Bund Naturschutz in Bayern e.V., Deggendorf, Germany; 6 Department of Environment and Health Studies, Telemark University College, Bø, Norway; 7 Scottish Society for the Prevention of Cruelty to Animals, National Wildlife Rescue Centre, Fishcross, United Kingdom; Sichuan University, CHINA

## Abstract

*Echinococcus multilocularis* is an important pathogenic zoonotic parasite of health concern, though absent in the United Kingdom. Eurasian beavers (*Castor fiber*) may act as a rare intermediate host, and so unscreened wild caught individuals may pose a potential risk of introducing this parasite to disease-free countries through translocation programs. There is currently no single definitive ante-mortem diagnostic test in intermediate hosts. An effective non-lethal diagnostic, feasible under field condition would be helpful to minimise parasite establishment risk, where indiscriminate culling is to be avoided. This study screened live beavers (captive, n = 18 or wild-trapped in Scotland, n = 12) and beaver cadavers (wild Scotland, n = 4 or Bavaria, n = 11), for the presence of *E*. *multilocularis*. Ultrasonography in combination with minimally invasive surgical examination of the abdomen by laparoscopy was viable under field conditions for real-time evaluation in beavers. Laparoscopy alone does not allow the operator to visualize the parenchyma of organs such as the liver, or inside the lumen of the gastrointestinal tract, hence the advantage of its combination with abdominal ultrasonography. All live beavers and Scottish cadavers were largely unremarkable in their haematology and serum biochemistry with no values suspicious for liver pathology or potentially indicative of *E*. *multilocularis* infection. This correlated well with ultrasound, laparoscopy, and immunoblotting, which were unremarkable in these individuals. Two wild Bavarian individuals were suspected *E*. *multilocularis* positive at post-mortem, through the presence of hepatic cysts. Sensitivity and specificity of a combination of laparoscopy and abdominal ultrasonography in the detection of parasitic liver cyst lesions was 100% in the subset of cadavers (95%Confidence Intervals 34.24–100%, and 86.7–100% respectively). For abdominal ultrasonography alone sensitivity was only 50% (95%CI 9.5–90.6%), with specificity being 100% (95%CI 79.2–100%). For laparoscopy alone sensitivity was 100% (95% CI 34.2–100%), with specificity also being 100% (95% CI 77.2–100%). Further immunoblotting, PCR and histopathological examination revealed one individual positive for *E*. *multilocularis*, whilst the other individual was positive for *Taenia martis*.

## Introduction

*Echinococcus multilocularis* is a zoonotic parasite of serious health concern, and regarded as one of the most pathogenic parasitic zoonosses in the Northern hemisphere (N. America, northern and central Eurasia) [1, 2]. Human infection presents as slow growing metacestodes, with similar behaviour as malignant neoplasia of the liver. It is usually fatal if untreated [3]. The mean annual incidence rates of *E multilocularis* infection in humans varies between 0.02 and 1.4 cases per 100,000 inhabitants in different European regions [4]. Red foxes (*Vulpes vulpes*) are the usual definitive host, but domestic dogs (*Canis lupus familiaris*) and racoon dogs (*Nyctereutes procyonoides*) are also highly susceptible to infection and parasite egg excretion [5, 6, 7], while cats (*Felis catus*), although becoming infected, rarely shed parasite eggs in their faeces. Although established in many countries across central Europe, other European countries are presently deemed free of this parasite, including the United Kingdom which employs strict measures to prevent entry, i.e. Pet Travel Scheme [8]. Recent studies have demonstrated that the range of *E*. *multilocularis* is more widespread than previously believed [9], with the parasite detected in red foxes in Sweden in 2009, an area where it was previously absent [10]. *E*. *multilocularis* prevalence in red foxes varies from 1 to 60% across different countries [1]. Infections with the metacestode stage of *E*. *multilocularis* have been observed in various domestic and wild animal species, with a range of microtine and arvicolid rodent species being the principle intermediate hosts [11, 12].

The Eurasian beaver (*Castor fiber*) is also a potential intermediate host. Once widespread across Europe and Asia, it was reduced to an estimated 1200 individuals by the beginning of the 20^th^ century [13], primarily through over-hunting for fur. Beavers previously occurred widely throughout Britain [14], but are thought to have become extinct in the 16^th^ century [13]. Conservation efforts have seen Eurasian beavers restored through translocations and reintroductions across much of its former native range [13, 15, 16]. Most recently this has included a Scottish Government sanctioned scientific trial reintroduction in Scotland (Scottish Beaver Trial). This translocation followed strict Office International des Epizooties (OIE) and International Union for Conservation of Nature (IUCN) guidelines on quarantine and health screening prior to release [17, 18]. Outside of this formal process it is now evident that a large population of beavers has become established throughout the River Tay and Earn catchments in Perthshire, Scotland [19]. This population is thought to have originated from escapes or illegal releases from captive collections. Recent genetic screening has demonstrated these are Eurasian beavers of Bavarian origin [20]. Feasibility reports for the release of beavers in England and Wales have also been developed [21, 22]. A Welsh pilot Eurasian beaver reintroduction is proposed [23] and there is now evidence of small numbers of wild beavers, again presumed escapes from captive collections, in England [24].

Barlow et al. [25] diagnosed *E*. *multilocularis* in a captive beaver at post-mortem. This individual was held in an English captive collection but had been directly wild-caught and imported years previously from Bavaria, Germany. *E*. *multilocularis* was first identified in Eurasian beavers from Switzerland [26, 27], and Austria [28], and more recently in Serbia [29]. Diagnosis of *E*. *multilocularis* in intermediate (non-egg-shedding) hosts such as beavers has historically been via post-mortem examination, with diagnosis in live hosts such as beavers being difficult and inconsistent. The occurrence of this positive individual has drawn significant attention to the potential risk posed by unscreened beavers and the establishment of *E*. *multilocularis* in Britain, although the likely risk has been evaluated as negligible [30].

Maintenance of *E*. *multilocularis*-free status is an important consideration in UK and Ireland. This is regarded as the primary reason for the continued use of the UK’s “pet passport” system and treatment of domestic dogs with praziquantel 1–5 days prior to re-entering the UK [31]. A risk assessment determined a 98% likelihood of at least 1 in 10,000 dogs returning to the UK from a short trip to Germany being infected with the parasite if untreated, and over 99% risk if dogs had been longer-term residents [3].

There is currently no single ante-mortem "gold standard" diagnostic test for *E*. *multilocularis* in intermediate hosts [32]. In human medicine, clinical diagnosis can be challenging, and relies on combinations of imaging modalities such as ultrasonography, computed tomograpy (CT), or magnetic resonance imaging (MRI), serology, nucleic acid detection, surgery, and histopathology [33].

A serological test could offer a non-invasive and low resource-intensive diagnostic tool for *E*. *multilocularis* in beavers, applicable for screening imported animals, those already present in captivity and potential source populations for conservation translocations [34]. Recent validation of differing serological tests in Eurasian beavers has determined immunoblotting, using a specific anti-beaver IgG conjugate, as the most reliable serological test, with a diagnostic sensitivity of 85% and specificity of 100% [34]. This sensitivity value could lead to false negatives and missing infected animals on screening. This makes the test more suited to secondary corroborative testing than primary diagnostic screening, particularly when there is a low prevalence of the disease in a population, as reported in beavers. While a useful diagnostic modality for assessing both live and dead beavers, this immunoblot test cannot be completed in real-time on live beavers in the field. Its current application in live-trapped beavers in the UK would require the animals to be maintained in captivity until results were available. Alternatively it would require the blood sampling then release of beavers, with subsequently re-trapping and dispatch required according to any proved positive results. This may prove difficult logistically and presents a risk of positive beavers being consumed by final host species such as red foxes before recapture. Specific to the screening of unlicensed wild-living beavers within Scotland was the requirement that any trapped and screened individuals were not held in captivity but re-released as soon after capture as possible [35]. This was seen as an acceptable compromise by the Tayside Beaver Study Group that represents stakeholders both for and against beaver reintroduction to Scotland, whilst the decision on full beaver reintroduction to Scotland is still being formally considered by the Scottish Government (Campbell-Palmer et al. 2015). A combination of real-time diagnostic testing methods, viable under field conditions, with later laboratory corroboration of results would appear to offer the best potential for the accurate non-lethal screening of beavers for *E*. *multilocularis*, in situations where this was specifically required.

The Royal Zoological Society of Scotland was approached to health screen both live captive and wild caught beavers for this parasite, with opportunistic post-mortem examinations performed on any cadavers found. Laparoscopic examination of the abdominal cavity for parasitic cysts was undertaken in combination with ultrasonography to allow the operator to visualize the parenchyma of organs such as the liver, or inside the lumen of the gastrointestinal tract, to determine cyst presence. Wild Scottish beavers were trapped under license from Scottish Natural Heritage, the conditions of which stated any beaver trapped and screened had to be released on the same day and at the point of capture, therefore temporary holding in captivity pending immunoblot results was not possible. The captive beavers screened in this study were trapped with permission of their owner for surgical neutering. Screening for *E*. *multilocularis* in these individuals was opportunistic whilst undergoing minimally invasive laparoscopic sterilization. These beavers were housed on a large, enclosed lake, making recapture, pending any positive immunoblot results, difficult but possible. This study was performed to investigate the usefulness of a combination of minimally invasive laparoscopy and abdominal ultrasound examination of the abdomen as a real-time screening diagnostic test under field conditions. The findings from this combination were then assessed against a validated immunoblot, PCR and post-mortem findings in a sample of beaver cadavers.

## Materials and Methods

This study was carried out in strict accordance with the Animal Welfare (Scotland) Act 2006, and all aspects were approved by the Royal Zoological Society of Scotland internal ethics committee, for overall study design and application. All wild animal trapping and veterinary examination within the Tayside region took place under license from Scottish Natural Heritage, license number 14529. Captive animals in Scotland and England were examined according to UK Veterinary Standards for private client examination at the owner’s request and with the owner’s permission. All Bavarian samples were collected through BUND (Bund Naturschutz de Bayern) beaver managers, with Bavarian state permission. All live animal surgery was performed under full general anaesthesia using isoflurane in oxygen, under veterinary supervision with full analgesia and all efforts were made to minimize suffering.

Three different groups of Eurasian beavers were included; a captive beaver family consisting of predominantly captive-born offspring from an imported wild caught pair from Bavaria, Germany (n = 18); wild-trapped animals (n = 12) and cadavers (n = 4) of unknown origin (captive bred or direct import) found across the River Tay and Earn catchments, Scotland; and shot wild Bavarian individuals in Germany (n = 11). Beavers of both sexes (female n = 21, male n = 24), and a variety of age classes ranging from kits and dispersers to breeding adults were examined, with weights ranging from 6.4–26.4kg (mean = 15.6 SD± 5.6kg).

### Study animals

#### Captive collection animals

Eighteen captive beavers of known Bavarian origin (directly imported breeding pair with captive born offspring), kept in a large enclosed lake on an English private estate were sterilised using minimally invasive surgery laparoscopic techniques, under field conditions in September 2011. This was done at the owners request and with their permission for animal management purposes. These beavers were trapped from a boat with a specifically designed landing net [36]. Two converted stables acted as temporary holding pens, pre- and post-surgery before re-release (24–48hrs in total). These beavers were monitored through visual observations daily for two weeks following release, then periodically for the following six months. For these individuals *E*. *multilocularis* investigation was via laparoscopic investigation and immunoblotting screening only.

#### Wild Scottish animals

These beavers were live trapped using Bavarian beaver traps (n = 12), then transported to the testing site for full health screening under anaesthetic. After health screening procedures were completed each animal was fully recovered from anaesthetic within a transport crate, they were passed suitable for transportation and re-release by the veterinary surgeon. Each beaver was re-released at point of capture, within 7hrs of examination. E. multilocularis investigation was via laparoscopic, ultrasound, and later immunoblotting screening investigation. Individuals were not specifically monitored post-release, though survival, normal behaviours and good body condition were observed opportunistically on some individuals via visual and camera trap observation, and one instance of clinical examination of a re-trapped individual [35].

In addition 4 cadavers were collected from various landowners. These individuals were either shot (n = 3) or had died in road traffic incidence (n = 1). Post-mortem examinations (standard methods with gross examination of all chest and abdominal organs, and histopathology performed), were undertaken within 2 days of collection, being kept refrigerated until the veterinary surgeon was available. *E*. *multilocularis* investigation was via post-mortem laparoscopy and ultrasound, followed by full post-mortem examination and immunoblotting screening investigation.

#### Wild Bavarian cadavers

All wild Bavarian cadavers (n = 11) were individuals removed as ‘problem’ beavers as part of the routine beaver management programme undertaken by southern Bavarian county administrations (BUND managers). They were dispatched by either a local administration veterinary surgeon (6 individuals) as performed routinely for other mammals using barbiturate parenteral drugs, or close range shot with .22 WMR in specific kill pens by BUND managers under license from the Bavarian state authorities.

### Screening methodology

#### Anaesthesia in live beavers

Anaesthesia in all live beavers was induced with isoflurane in oxygen, administered via a facemask. Beavers were then intubated using 4.5–6.0mm internal diameter, high volume, low pressure PVC endotracheal tubes (PORTEX). Beavers were maintained with spontaneous ventilation with isoflurane (IsoFlo, Abbot Laboratories Ltd) and oxygen on a Bain or a Modified Ayres T-Piece anaesthesia system as size dictated. Anaesthesia was additionally monitored in some cases with side-stream capnography and combined pulse oximetry (VM-2500, Viamed), arterial blood gases (EPOC, Woodley Equipment Ltd), and rectal/oesophageal thermometer probe (International Animal Rescue). For each individual the feet and tail were wrapped in aluminium tin foil to prevent body heat loss during anaesthesia [37].

#### Blood sampling

In all live individuals blood was collected aseptically from the tail vein for haematology and serum biochemistry to assess individuals’ general state of health (SAC Consulting Veterinary Services, Scottish Rural College). Samples were also taken (EDTA tubes) for diagnostic testing via immunoblotting (University of Bern) [34]. For cadavers blood was collected from the heart or, if blood was too clotted, from peripheral vessels and sent for immunoblotting testing.

#### Abdominal ultrasonography

Abdominal ultrasonography directed specific attention to the liver for any cystic structures that could be suspicious for *E*. *multilocularis*. A 3.5–5MHz frequency convex abdominal ultrasound probe was used (BCF Ltd), and examinations recorded. Ultrasonography was performed by wetting the dense fur with 90% ethanol to allow adequate contact and visualisation, in preference to fur-clipping, which may have adversely affected water-proofing and thermal insulation when beavers are returned to the wild after testing.

#### Laparoscopic examination

Whilst under anaesthetic a full laparoscopic examination of the liver and other abdominal organs was undertaken for any visible internal lesions that could be suspect for *E*. *multilocularis* infection. For the captive individuals, a small strip of fur was clipped over the ventral midline, between the pubis and umbilical scar, limited in size to less than 10–14cm in length and 1–2cm width to reduce the effects on waterproofing and body temperature maintenance after surgery. These animals were also undergoing laparoscopic sterilisation which required an additional instrument port to be placed, hence the increased area of clipped fur. This area of clipped fur was reduced to ~1cm square region in later individuals, however, following this the majority of individuals had no fur clipping performed to limit loss of water proofing and thermal insulation in their aquatic environment. The fur and skin in the ventral midline region of the umbilicus was thoroughly cleaned and disinfected with a chlorhexidine (0.05%) or povidone/iodine (0.02%) based surgical scrub, followed by the application of surgical ethanol (80%). Before surgery 50 mg/kg long acting amoxicillin (Depocillin, Intervet UK Ltd) and 0.3mg/kg meloxicam (Metacam, Boehringer Ingleheim) was administered by subcutaneous injection.

Beavers were positioned in dorsal recumbency for the initial general laparoscopic examination. A 4mm skin incision was made and the underlying ventral muscles blunt dissected to allow open access placement of a blunt trocar and 3mm or 5mm cannula (YelloPort Surgical Innovations) depending on body size. This was placed caudal to the umbilical scar, approximately one-third of the distance between the umbilical scar and pubis. The abdomen was insufflated with 8–10 mmHg pressure using medical grade carbon dioxide. The abdomen was initially examined with a 3mm or 5mm, 30 degree, 30cm paediatric laparoscope (Karl Storz UK). The animal was repositioned in left and right lateral recumbency to allow movement of the viscera, and visualisation of all organ surfaces. Additional cannulas, 3mm palpation probe, 3mm atraumatic Rothernburg forceps, and 3mm biopsy forceps (Karl Storz UK), were available, should they be required for manipulation or sampling of tissues.

**Fig 1:**
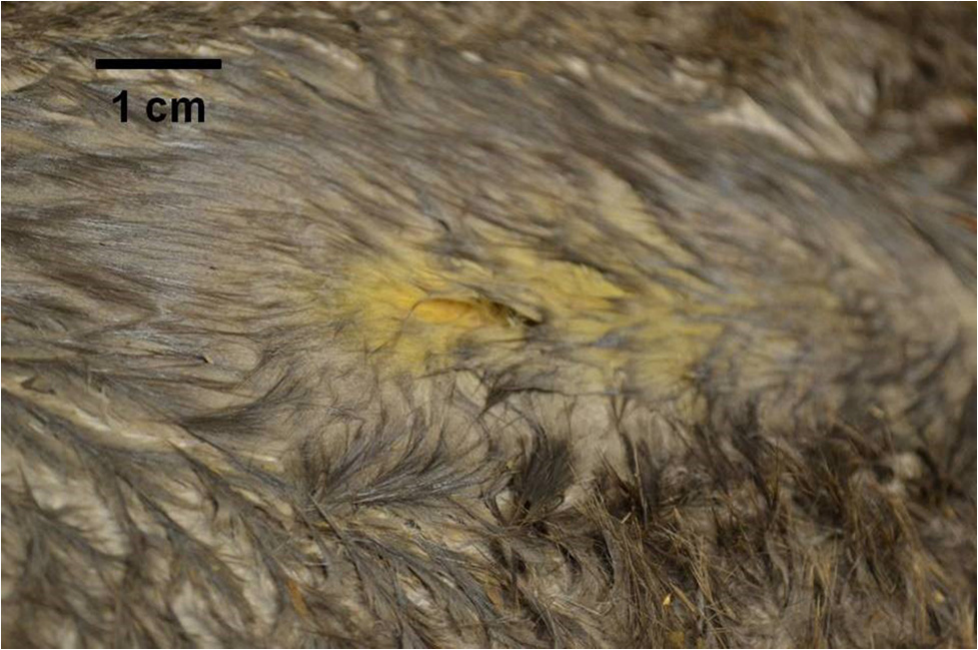
Post laparoscopic investigation incision site. View of incision site on closing following laparoscopic insertion, yellow stain marks represent iodine solution as fur wetted to aid ultrasound examination.

The abdomen was examined in a standardised manner starting with the dorsal and ventral surfaces of all the liver lobes and gallbladder, falciform ligament and peritoneal surface, stomach surface, spleen, small intestine, mesentery and lymph-nodes, inguinal canals, bladder, and pelvic entrance. Beavers were then manually tilted into alternating lateral recumbency to allow examination of the lateral and dorsal abdominal walls, kidneys, adrenal glands, and ovaries and uterine horns in females. Port site wounds were closed in two layers with a single intramuscular suture of 1.5 metric poliglecaprone (Monocryl Ethicon), and an intradermal suture of 1.5 metric poliglecaprone (Monocryl Ethicon), with an Aberdeen knot buried beneath the skin. A thin layer of cyanoacrylate tissue adhesive was applied to the closed skin wounds (Gluture Abbott Laboratories Ltd). The resultant wound was approximately twice the size of a standard microchipping wound (Fig 1). Recovery was in a travel crate with deep straw layer.

#### Post mortem examination, histopathology and polymerase Chain Reaction (PCR)

Full post mortem examination was undertaken for wild Scottish cadavers (n = 4) and wild shot Bavarian individuals (n = 11). All hepatic cysts detected were sampled and fixed in 10% neutral buffered formalin, fixed for at least 24hours, and processed for histology. For PCR testing, genomic DNA samples obtained from two beaver specimens (Bavarian wild) were processed by the method described by Dinkel et al. [38]. Sequence data for the PCR-generated fragment of the mitochondrial 12S rRNA gene [38] were used to screen Genebank for similarities/identities.

All examinations undertaken are summarized in Table 1.

**Table 1:** Beaver category with screening method employed.

	Cadaver		Live trapped*		Total
Screening Method	Bavarian wild (n = 11)	Scottish wild (n = 4)	Scottish wild (n = 12)	Captive collection (n = 18)	
Laparoscopic	11	4	12	18	45
Ultrasound	11	4	12	*na*	27
Post-mortem	11	4	*na*	*na*	15
Immunoblotting	2	4	12	18	36

Frequency of individual tests for the detection of cysts and *Echinococcus multilocularis* associated hepatic lesions.

*na* = not examined

* also tested for haematology and serum biochemistry

### Statistical analysis

Statistical analysis were undertaken in WinPepi, Version 11.43 using the DESCRIBE 2.68 program, with prevalence calculated using EpiTools [39]. For combinations of tests, a parallel testing approach was used (i.e. a positive screening in any or both of the tests combined equates to a positive diagnosis, and a negative result in both tests is necessary for a negative result in the combination test).

## Results

### Examinations

**Fig 2:**
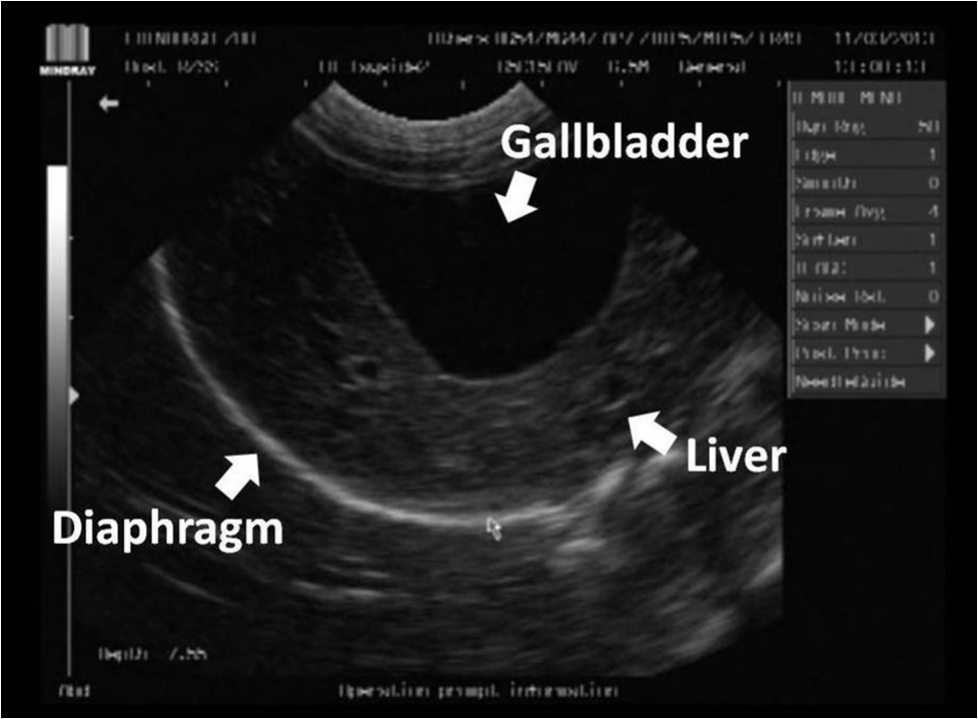
Normal sonogram. Ultrasound image of normal abdominal organs in an anaesthetised beaver.

**Fig 3:**
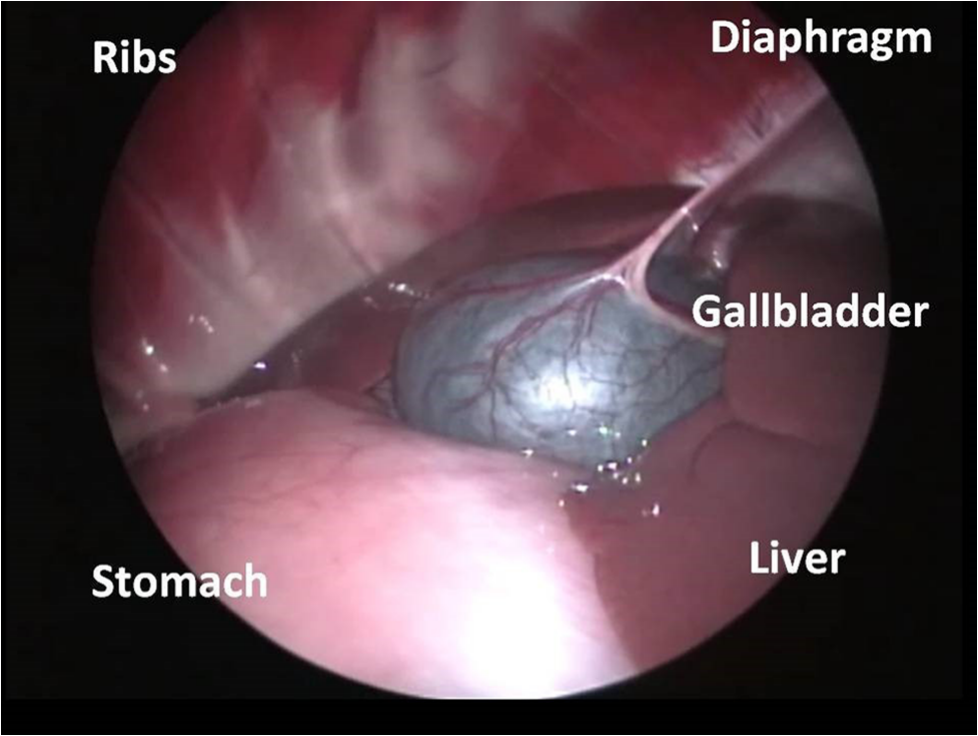
Normal laparoscopic image. Laparoscopic image of normal abdominal organs in an anaesthetised beaver.

This study evaluated *E*. *multilocularis* screening methods in three separate beaver groups; randomly sampled wild Scottish beavers (live and cadavers), randomly sampled Bavarian beavers (cadavers), and a captive beaver family (live). All live beavers screened were largely unremarkable in their haematology and serum biochemistry with no values suspicious for liver pathology or potentially indicative of *E*. *multilocularis* infection. This correlated well with ultrasound (Fig 2), laparoscopy (Fig 3), and immunoblotting, which were unremarkable in all live individuals.

**Fig 4:**
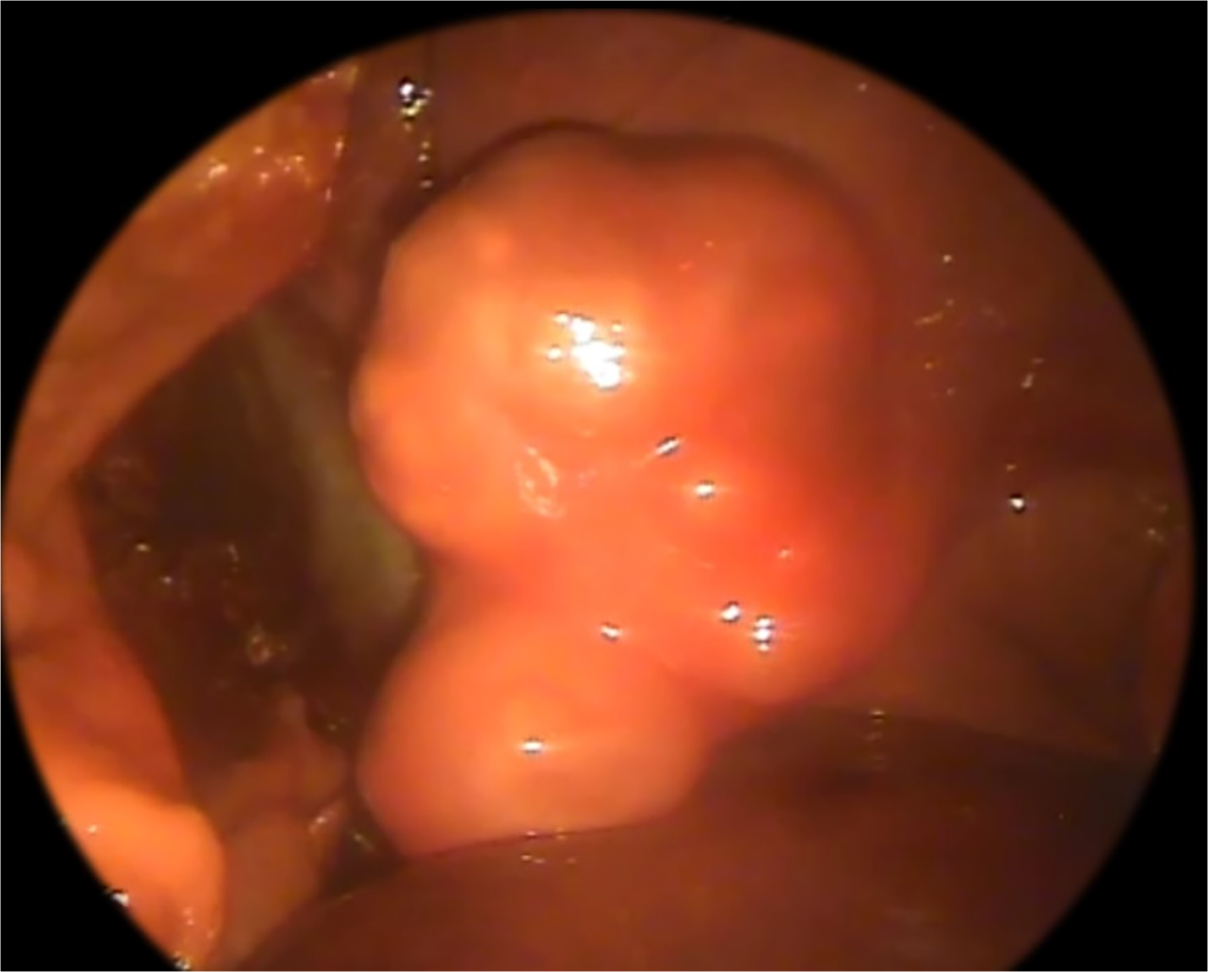
Laparoscopic view of *E. multilocularis* cyst. Laparoscopic view of *E*. *multilocularis* infection in the liver of a beaver cadaver.

**Fig 5:**
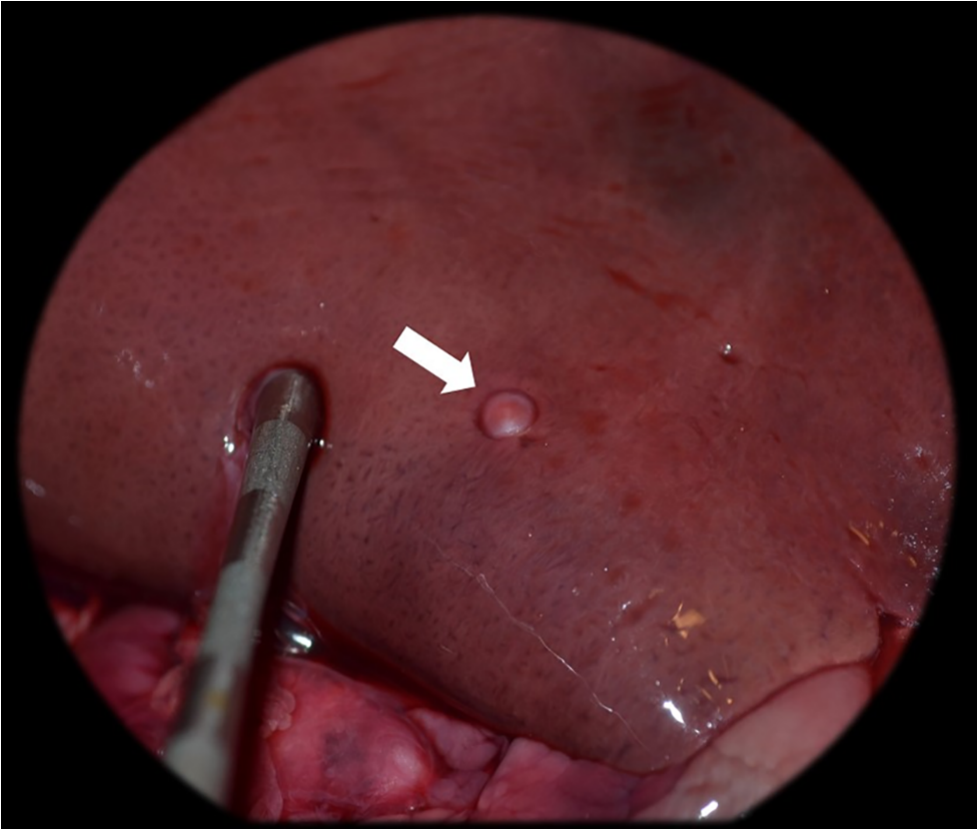
Laparoscopic view of a *Taenia martis* cyst. Laparoscopic view of a small *Taenia martis* cyst in a beaver cadaver liver, with laparoscopic palpation probe with 10mm graduations).

**Fig 6:**
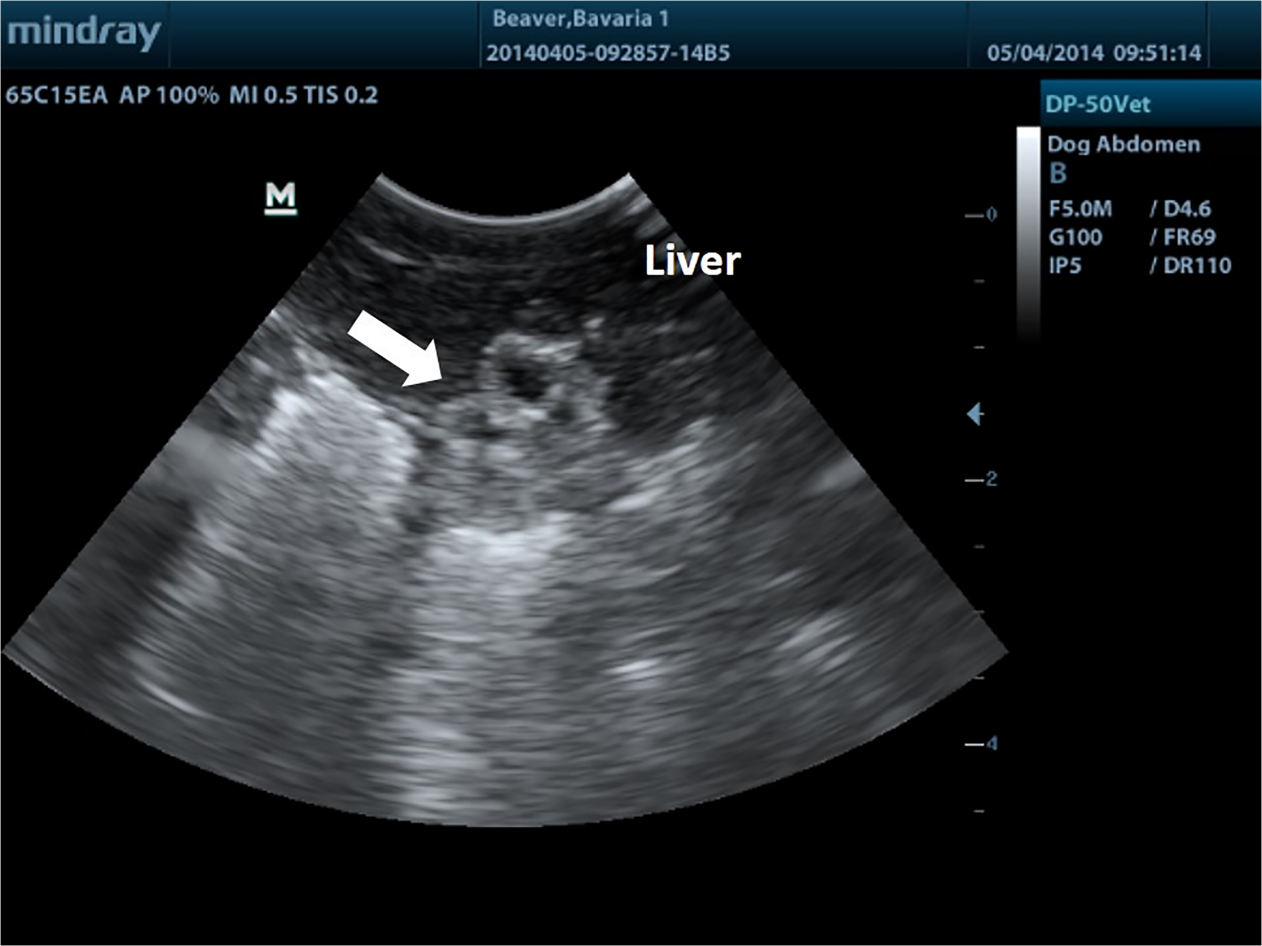
Sonogram of *E*. *multilocularis* cyst. Ultrasound image of *E*. *multilocularis* cyst in the liver of the same beaver cadaver as Fig 4.

**Fig 7:**
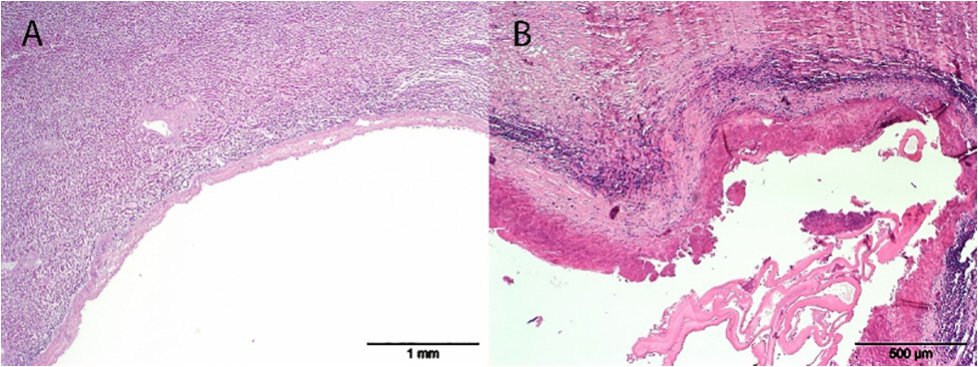
Histological presentation of *E*. *multilocularis*. Histological presentation of a 25mm wide hepatic cyst, consistent with *Echinococcus multilocularis* in PCR analysis. Section A: This cyst is encapsulated by a band of fibrous tissue, and features a mostly empty lumen. Section B In some areas, the lumen contains hypereosinophilic, hyaline membranes, and mild mineralization of the inner aspect of the capsule. Protoscolices were not noted in any of the sections examined.

**Fig 8:**
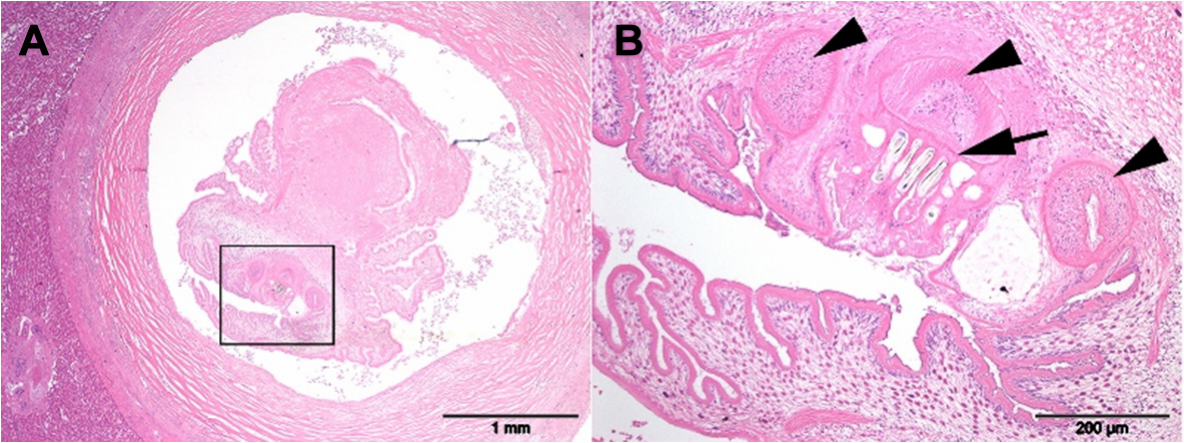
Histological presentation *Taenia martis*. Histological presentation of 3mm wide hepatic cyst, consistent with *Taenia martis* in PCR analysis. Section A: The cyst is lined by a band of thick fibrous tissue, and contains an intraluminal, 2mm wide metazoan organism. Section B (inset of Section A): This organism does not possess a coelomic cavity and features a protoscolex (arrow), and three suckers (arrowheads). H&E stain.

Most cadavers presented similar results, with the exception of two individuals. These were both wild-shot Bavarian beavers which had cystic lesions in the liver on postmortem examination. These cases were also detected through previous laparoscopy (Figs 4 and 5), whilst ultrasonography only detected one case (Fig 6). Further testing of these cystic lesions was undertaken with histology, immunoblotting and PCR. Testing by PCR and immunoblotting revealed one *E*. *multilocularis* positive sample (this lesion had been detected both ultrasonographically and laparoscopically). Histologically this lesion was composed of a thick, multifocally mineralized, encapsulated cyst, containing hyaline membranes and granular eosinophilic material (Fig 7). Protoscolices were not noted in the section examined, which may have resulted as an artefact from freezing (through membrane breakage and loss of morphological detail) or potentially due to angle of the cut of the section. Conversely, the samples from the second beaver with liver cysts were negative with immunoblotting and positive for PCR. This cystic lesion had been detected only laparoscopically. Sequence analysis of the PCR amplicon revealed a 99% sequence identity with *Taenia martis*. Histologically this lesion was composed of an encapsulated cyst containing a single cysticercus with an invaginated protoscolex (Fig 8).

### Test evaluation

The sensitivity and specificity of abdominal ultrasound and laparoscopic examination in the detection of parasitic cystic lesions that could be associated with *E*. *multilocularis* was determined by standard diagnostic evaluation methods in the cadaver sample (n = 15). The gold standard was the outcome of postmortem, histology, immunoblotting and PCR tests. For laparoscopic examination, (two positives), the sensitivity was 100% (95%CI = 34.2–100%) and specificity 100% (95%CI = 77.2–100%). For ultrasound examination alone (one true positive and one false negative) the sensitivity was 50% (95%CI = 9.5–90.6%), and a specificity of 100% (95%CI = 79.2–100%). The combined approach (laparoscopy and abdominal ultrasound) had a sensitivity of 100% (95%CI = 34.2–100%) and a specificity of 100% (95%CI = 86.7–100%).

### Prevalence estimation

The combination of laparoscopy and abdominal ultrasound was used to estimate the prevalence of *E*. *multilocularis* in the populations studied. This prevalence was zero in both the captive collection (n = 18; 95%CI = 0–0.18) and the wild Scottish population (n = 16; 95%CI = 0–0.19). The prevalence of hepatic cysts in the Bavarian wild shot population was 0.18 (n = 11; 95%CI = 0.05–0.48), of the two instances of hepatic cysts, one was confirmed as *E*. *multilocularis* which results in a prevalence of 0.09 (n = 11; 95%CI = 0.02–0.38).

## Discussion

Several authors have highlighted that unlicensed releases of Eurasian beavers may pose a potential risk of introduction and establishment of *E*. *multilocularis* in currently disease free countries such as the United Kingdom [40, 41, 42]. While qualitative risk assessments deemed this risk as low to negligible [8, 30], it is not possible to accurately quantify the actual risk without further studies. It is recommended that beaver cadavers of directly imported (or potentially imported animals) are examined for the presence of the parasite. Strict adherence to OIE and IUCN guidelines on the quarantine and health screening of all beavers prior to importation is advised [17], best practice would also see that beavers are not directly imported from *E*. *multilocularis* endemic countries, including Bavaria [8], which has historically seen a large number of wild caught beavers exported to other countries. Eurasian beavers released as part of the SBT were imported from Norway where this risk is considered negligible [10, 43]. However, *E multilocularis* has recently been found in Sweden in two foxes less than 65km from the Norwegian border [44], and it is possible that in time the Norwegian population, particularly eastern Norwegian, beavers may cease to be regarded as *E*. *multilocularis* free. Therefore sourcing of animals for future releases in disease free countries should ideally be from regions free of this and other notable diseases, or by only releasing second generation captive bred individuals.

Current available ante-mortem diagnostic testing modalities for *E*. *multilocularis* (immunoblotting, post-mortem, laparoscopic, ultrasound examinations), all have limitations if used on their own in any intermediate host species. Serum biochemistry tests may demonstrate elevations in liver enzymes, due to liver damage caused by any parasitic cystic lesions, but these may be relatively insensitive and non-specific tests on their own and have not been validated for use in diagnosis of *E*. *multilocularis* in beavers. This study found that laparoscopic examination provided excellent visualization of the abdomen and detailed viscera examination, whilst being relatively minimally invasive. Laparoscopy is not on its own a definitive test for *E*. *multilocularis*, and does not allow the operator to visualize any small potential cysts inside the parenchyma of organs such as the liver, or inside the lumen of the gastrointestinal tract, hence the advantage of its combination with abdominal ultrasonography. Recovery in live beavers was rapid and unremarkable, although a single individual was lost on recovery from anaesthetic. This technique appears a viable useful adjunct diagnostic technique in the field for real-time screening of live beavers for *E*. *multilocularis* infection if captive care is not possible.

The recent evaluation of immunoblotting as having a diagnostic sensitivity of 85% and specificity of 100% in the diagnosis of *E*. *multilocularis* in Eurasian beavers, offers a useful testing method, particularly for captive held animals or investigation of source population prevalence [34]. The high specificity means that when the test is positive, it is almost certain the animal is indeed infected with *E*. *multilocularis*. However, with a sensitivity of 85% means there is a chance of false negatives where infected individuals test negative on the immunoblot. Given the consequences of *E*. *multilocularis* not being detected when it is actually present, this could be cause for concern in a screening test, particularly in countries where this parasite is absent and the reintroduction of beavers may pose a low potential risk (defined as rare but does occur) [30]. For this reason it is most useful as a corroborative test, used in conjunction with other diagnostic modalities for screening.

Minimally invasive surgical techniques such as laparoscopy, hold notable aadvantages over open abdominal surgery in animals. These advantages include small wounds, rapid recovery, minimal post-operative pain, rapid wound healing, low rates of infection, and low risk of wound dehiscence [45, 46, 47, 48, 49]. In wild animals these advantages allow a rapid return to the wild, and also a rapid return to an aquatic environment, which is important in beavers. Laparoscopy also provides magnified visualisation of organs, particularly in parts of the abdomen difficult to visualize in open surgery. When performed by an experienced surgeon the abdominal organs can be better and more thoroughly examined than with open abdominal surgery [50, 51]. Abdominal surgery in free-ranging wild animals carries a well reported risk of serious complications and death in individuals, greater than that in domestic animals [52, 53, 54]. This was avoided in this study with the use of minimally invasive surgical technique. Previous studies involving abdominal surgery in free-ranging Eurasian beavers have reported a single mortality [55].

Laparoscopic screening revealed two individuals (both shot wild Bavarian beavers) that had suspect liver lesions. Further immunoblotting and/or PCR should be employed to determine parasite identification, if management protocols do not permit captive holding of beavers until processing of results then laparoscopic examination may permit differentiation based on lesions structure in some cases. *E*. *multilocularis* cysts are multicystic whereas *Taenia* spp. for example are singular cysts. It should be noted this may not be possible to detect in early infestation. Ultrasonography was performed on 27 individuals. This testing modality on its own missed one individual with parasitic liver cystic lesions (sensitivity 50%), which were subsequently seen on both laparoscopic and post-mortem examination. The limitation of this diagnostic modality is partly due to the beaver’s voluminous gastrointestinal tract containing gas and ingesta, resulting in the potential that the entire liver and all abdominal may not be able to be completely visualised [42]. Similar limitations have been found in sheep (*Ovis aries*) in which ultrasound examination for cystic echinococcosis gave a test sensitivity of ~89% and a specificity of 76% [56].

Laparoscopy and ultrasound will not be able to diagnose very early cases of infection, i.e. the time between oral infection and the development of visible lesions, the same is true for post-mortem examination. In such circumstances immunoblotting may provide the most likely method of any positive diagnosis, whilst laparoscopy, ultrasound and post-mortem examination are all likely to result in a negative diagnosis, even in infected individuals. With an 85% sensitivity the immunoblotting may also miss an infected animal or misdiagnose a negative animal as positive. While no single current diagnostic test is definitive, the combined use of laparoscopic and ultrasound investigation for real-time diagnosis of *E*. *multilocularis* in beavers under field conditions will allow the direct rapid identification of any abdominal lesions. Additionally submission of blood samples for immunoblotting should be undertaken to identify any suspect cysts and potentially determine animals that are serologically positive, including earlier stage infections which are then likely to develop more obvious lesions in the future.

While there is an anecdotal report of a prevalence of 2.5–5% *E*. *multilocularis* infection in Eurasian beavers in Bavaria, based on hunter’s recall from 400 culled beavers [25], the actual prevalence of *E*. *multilocularis* is unknown. It is hence impossible to accurately quantify the risk beavers pose in establishing *E*. *multilocularis* in disease free regions without further studies. The low prevalence of a disease found in this study considerably increases the risks of misdiagnosis with any single test. Relating this to beaver management in Britain, for example, means the very low likely prevalence rates may increase the chances that any individuals testing positive may not actually be carrying *E*. *multilocularis* and so maybe culled due to a false positive screening result.

While further work would help validating the usage of the combined testing undertaken, currently the technique appears to provide a viable real-time screening method in the field, as an alternative to culling beavers in efforts to minimizing the risk that beavers of unknown origin pose in establishing *E*. *multilocularis* in the UK. Laparoscopy combined with ultrasound may also help in minimizing the risk posed by any beavers imported for future reintroductions from *E*. *multilocularis* positive regions.
